# Perioperative Challenges of Heroin Addiction: A Case Report of Opioid-Free Anesthesia in Tongue Carcinoma Excision With Free-Flap Reconstruction

**DOI:** 10.7759/cureus.41195

**Published:** 2023-06-30

**Authors:** Roberto J Ameiro, Sara S Neves, Rita P Oliveira, Beatriz B Marques, Paulo-Roberto C Ferreira

**Affiliations:** 1 Department of Anaesthesiology, Centro Hospitalar de Vila Nova de Gaia/Espinho, Vila Nova de Gaia, PRT; 2 Department of Anaesthesiology, Centro Hospitalar Universitário de Santo António, Porto, PRT

**Keywords:** free flap reconstruction, opioid abuse, perioperative pain management, heroin abuse, opioid-free analgesia

## Abstract

Anesthesia for major head and neck surgery is historically heavily reliant on opioids with deleterious consequences. We reported a case of a patient with a history of heroin abuse submitted to a tongue carcinoma excision, followed by free-flap reconstruction under opioid-free anesthesia. We used a propofol total intravenous anesthesia and perfusions of ketamine, dexmedetomidine, lidocaine, and magnesium sulfate for analgesia, complemented by boluses of dexamethasone, acetaminophen, parecoxib, and metamizole. Hemodynamic needs of the procedure were addressed by titrating perfusions of sodium nitroprusside or dobutamine. The patient was weaned from the ventilator at the end of the surgery. Surgical outcomes were achieved and opioid-free analgesia allowed early reestablishment of bodily functions without compromise of adequate pain control. Anesthesia protocols for free-flap surgery still lack scientific evidence, especially in the context of substance abuse: opioid-sparing approaches seem a viable option, which requires further studies and familiarity by health care professionals.

## Introduction

The use of strong opioids in the community has risen dramatically in the past decades, representing a burden on health institutions. Anesthesiologists can come across chronic opioid users in multiple contexts of the perioperative period, and clinical management raises multiple challenges. These patients have increased perioperative analgesic requirements, specifically when using opioid regimens, resulting in a higher incidence of side effects, an increase in hospital length of stay, and financial costs [1].

Opioid-free anesthesia has been defended by some authors as the new paradigm of perioperative care. Some institutions and individuals have developed drug protocols for opioid-free anesthesia, but there are surprisingly few clinical trials and high-quality prospective studies that demonstrate the significant benefits of this type of anesthesia over conventional regimens [2].

## Case presentation

A 55-year-old female, American Society of Anesthesiologists (ASA) physical status 3, with squamous cell carcinoma of the tongue was scheduled for vocal cord lesion excision, glossectomy, and segmental mandibulectomy. A 3D-printed bone frame was used to fill in the defect, followed by tissue free-flap reconstruction.

The patient’s history was only significant for heroin and cocaine abuse currently enrolled in a methadone program. A preoperative physical exam revealed a partial obstruction of the oral cavity by the tumor. On the day of the procedure, the patient was monitored with ASA standards, neuromuscular blockade monitoring (train of four counts and *ratio*), anesthetic depth (Bispectral Index, BIS®), hourly urinary output, and invasive blood pressure. Due to the possibility of a difficult airway, airway and emergency equipment was accessible. Otorhinolaryngologists were present in the event of a necessary emergent tracheostomy.

The patient was pre-oxygenated, and intravenous anesthesia was initiated with propofol administered by target-controlled infusion (TCI) with Schnider’s pharmacokinetic model (effect site target of 2-4 µg/mL). Ketamine (0.1-0.25 mg/kg/h) and dexmedetomidine (0.8-1 mcg/kg/h) were also infused and titrated to obtain unconsciousness while preserving spontaneous ventilation. The airway was then inspected with a Macintosh blade as atraumatically as possible. Plan A was to secure it with a microlaryngoscopy orotraqueal tube using an AirTraq® device. Plan B included fibroscopy-guided intubation, and Plan C was to tracheostomize the patient. Plan A was successful at the first attempt, after which the patient was given rocuronium 0.6 mg/kg and mechanically ventilated on pressure-control mode, targeted to lung-protective volumes. 

After the vocal cord mass was excised, the patient was electively tracheostomized, and the intraoperative airway was secured with a laryngectomy tube.

Analgesia was approached in a multimodal and preemptive manner, consisting of lidocaine (loading dose 1.5 mg/kg, followed by an infusion 1.5 mg/kg/h), magnesium sulfate (loading dose 50 mg/kg, followed by an infusion 15 mg/kg/h), dexamethasone (8 mg), acetaminophen (1 g), parecoxib (40 mg), and metamizol (2 g).

We followed a goal-directed fluid therapy, aiming for a urinary output of at least 0.5 mL/kg/h. Concerning blood loss, we guided our approach according to patient blood management recommendations: we administered iron carboxymaltose (500 mg) the previous day and tranexamic acid (1 g) at the beginning of the procedure and used a transfusional policy aiming for hemoglobin of at least 8.0 g/dL.

Our strategy was to aim for relative hypotension to minimize blood losses during the first stage of surgical dissection - for this purpose, we also used a sodium nitroprusside infusion (0.3-1 mcg/kg/min). After flap anastomosis, our strategy was to increase its perfusion by increasing cardiac output while minimizing vasoconstriction - for this, we used a dobutamine infusion (2 mcg/kg/min). The intraoperative period had brief occasional episodes of hemodynamic lability, which rapidly reverted by changing the infusion rates of sodium nitroprusside and dobutamine. Lysine acetylsalicylate 900 mg was administered immediately before the completion of the anastomosis to decrease turbulent-flow-induced platelet aggregation *in situ*. The procedure lasted a total of 12 hours, and at the end, we reversed neuromuscular blockade with Sugammadex® (4 mg/kg, guided by neuromuscular blockade monitoring). The patient was transferred to the intensive care unit - conscious, tracheostomized, and on spontaneous ventilation. Vital signs varied within the expected range, with hemodynamic parameters titrated to enhance flap perfusion. Postoperative pain relief strategy included an infusion of dexmedetomidine (0.4 mcg/kg/h) and patient-controlled intravenous analgesia (PCA) of ketamine (2.5 mg/mL) and midazolam (0.5 mg/mL), set as perfusion of 1 mL/h and bolus on-demand of 0.5 mL (lock-out time 10 minutes), alongside acetaminophen (1 g 4i.d.), parecoxib (40 mg 2i.d.), and metamizole (2 g 2i.d.). Enteric diet was also initiated 12 hours postoperatively via gastrostomy with good tolerance, as well as methadone treatment. Weaning from dexmedetomidine and PCA was initiated in the first 24 hours, and the patient was transferred to the ward after 72 hours. There were no reports of moderate-to-severe pain, nor nausea/vomiting in the postoperative period. Surgical outcomes were successful, and the patient demonstrated a high level of satisfaction with anesthesia care.

## Discussion

Anesthetic management of a chronic opioid consumer includes dealing with acute and chronic tolerance to opioids, hyperalgesia phenomena, withdrawal reactions, and preventing relapse. Opioid-based anesthetic care has been the standard option for anesthesiologists for free flap surgery from very early on: opioids have a rapid onset of action, have great short-term efficacy, are cheap and available, and have no analgesic ceiling, explaining their wide use in the perioperative context. This does not come without a cost, as they also present with many adverse side effects, which are multisystemic: respiratory depression, leading to delay of ventilator weaning, and increased incidence of postoperative nausea and vomiting, delaying postoperative enteral diet introduction. Using opioids in high doses or for long periods is also associated with persistent opioid consumption months to years after surgery, increasing substance abuse rates [3,4]. There are also concerns about their role in immunosuppression and what that can mean in terms of oncologic surgery, with conflicting literature pointing out that they could increase disease relapse [5]. 

This particular surgery raised many anesthetic challenges: airway safety, respiratory morbidity, hemodynamic requirements, hemostatic peculiarities, analgesic requirements, the need for adequate monitoring to allow goal-directed therapies, and lastly *frailty *of the patient. The intraoperative period was uneventful, and the patient woke up on spontaneous ventilation with minimal sedation. She initiated enteral feeding as soon as possible, and the pain was adequately controlled without excessive sedation or risk of respiratory depression, even in the ward.

We believe that this case is important because it demonstrates that opioid-free anesthesia can be both safe and effective. To our knowledge, there is not any published work to date showing the use of this technique in major head and neck surgery involving microvascular anastomosis in populations such as opioid addicts.

## Conclusions

Anesthesia for major head and neck oncosurgery and free-flap surgery lacks satisfactory evidence to guide clinical practice, especially in peculiar populations, such as opioid addicts. It seems reasonable that an opioid-free approach can answer this challenge, but further studies are necessary. We believe case reports like this are vital to help expand knowledge in the matter and promote discussion of protocols.
